# Autoreceptor control of serotonin dynamics

**DOI:** 10.1186/s12868-020-00587-z

**Published:** 2020-09-23

**Authors:** Janet Best, William Duncan, Farrah Sadre-Marandi, Parastoo Hashemi, H. Frederik Nijhout, Michael Reed

**Affiliations:** 1 Department of Mathematics, The Ohio State University, 231 W 18th Ave., Columbus, OH 43210 USA; 2 Department of Mathematics, Duke University, Durham, NC 27708 USA; 3 qPharmetra, LLC, Denver, CO 80203 USA; 4 Department of Bioengineering, Imperial College, London, SW7 2AZ UK; 5 Department of Biology, Duke University, Durham, NC 27708 USA

**Keywords:** Serotonin, Autoreceptor, Mathematical model

## Abstract

**Background:**

Serotonin is a neurotransmitter that has been linked to a wide variety of behaviors including feeding and body-weight regulation, social hierarchies, aggression and suicidality, obsessive compulsive disorder, alcoholism, anxiety, and affective disorders. Full understanding involves genomics, neurochemistry, electrophysiology, and behavior. The scientific issues are daunting but important for human health because of the use of selective serotonin reuptake inhibitors and other pharmacological agents to treat disorders. This paper presents a new deterministic model of serotonin metabolism and a new systems population model that takes into account the large variation in enzyme and transporter expression levels, tryptophan input, and autoreceptor function.

**Results:**

We discuss the steady state of the model and the steady state distribution of extracellular serotonin under different hypotheses on the autoreceptors and we show the effect of tryptophan input on the steady state and the effect of meals. We use the deterministic model to interpret experimental data on the responses in the hippocampus of male and female mice, and to illustrate the short-time dynamics of the autoreceptors. We show there are likely two reuptake mechanisms for serotonin and that the autoreceptors have long-lasting influence and compare our results to measurements of serotonin dynamics in the substantia nigra pars reticulata. We also show how histamine affects serotonin dynamics. We examine experimental data that show very variable response curves in populations of mice and ask how much variation in parameters in the model is necessary to produce the observed variation in the data. Finally, we show how the systems population model can potentially be used to investigate specific biological and clinical questions.

**Conclusions:**

We have shown that our new models can be used to investigate the effects of tryptophan input and meals and the behavior of experimental response curves in different brain nuclei. The systems population model incorporates individual variation and can be used to investigate clinical questions and the variation in drug efficacy. The codes for both the deterministic model and the systems population model are available from the authors and can be used by other researchers to investigate the serotonergic system.

## Background

In 2010, three of the authors (JB, HFN, MCR) created a mathematical model of serotonin synthesis in varicosities, storage in vesicles, release into the extracellular space, reuptake by serotonin transporters (SERTs), and control by serotonin autoreceptors [1]. In subsequent years, they used the model to study and evaluate various hypotheses about serotonergic function including connections with dopaminergic signaling [2, 3], bursts in the dorsal raphe nucleus (DRN) [4], the effects of serotonin on levodopa therapy [5], and serotonin dynamics in the basal ganglia [6]. In 2013, they began a collaboration with Parastoo Hashemi, an electrochemist who can measure serotonin and histamine in the extracellular space in vivo in various brain regions of the mouse after stimulation of the DRN. This collaboration led to new insights into serotonergic function [7–9]. It also revealed that various aspects of the 2010 model were naive and too simplistic. This paper presents a new, substantially different, revised model.

In the experiments in the Hashemi Lab, the medial forebrain bundle (MFB) is stimulated for 2 s and the antidromic spikes excite the DRN. The DRN sends bursts of action potentials to projection regions such as the substantia nigra pars reticulata (SNr), the pre-frontal cortex (PFC), and the hippocampus (HC). Serotonin rises rapidly in the extracellular space in the projection regions and then typically plunges substantially below basal levels within 30 s [7]. This almost certainly is because inhibition of release by the autoreceptor continues well after the serotonin concentration in the extracellular space has returned to basal levels. In our 2010 model, the autoreceptor effect was modeled by high extracellular serotonin instantaneously inhibiting release, and the Hashemi experiments showed that this is wrong. In this paper, we include a full model of the cellular dynamics caused by serotonin binding to the autoreceptor, including activated receptor G-proteins and activated regulators of G-proteins. In addition, we showed in [8, 10] that histamine in the extracellular space inhibits the release of serotonin from serotonin varicosities. Therefore in this paper we also include a full model of a histamine $H_3$ receptor on the serotonin varicosity that changes the dynamics of serotonin release. We also make stochastic systems population models from our deterministic model (see below) and use these systems population models to investigate certain aspects of the serotonin system.

In [7], we also showed that there are two different serotonin uptake mechanisms, SERTs that pump serotonin back into the varicosities and another uptake, which we call Uptake 2, that pumps serotonin into glial cells [11–13]. The kinetics of the two uptakes are quite different and both are included in our new model. We also include the effects of serotonin binding protein (SBP) that binds serotonin tightly in vesicles but releases it quickly when the vesicles open to the extracellular space.

**Fig. 1:**
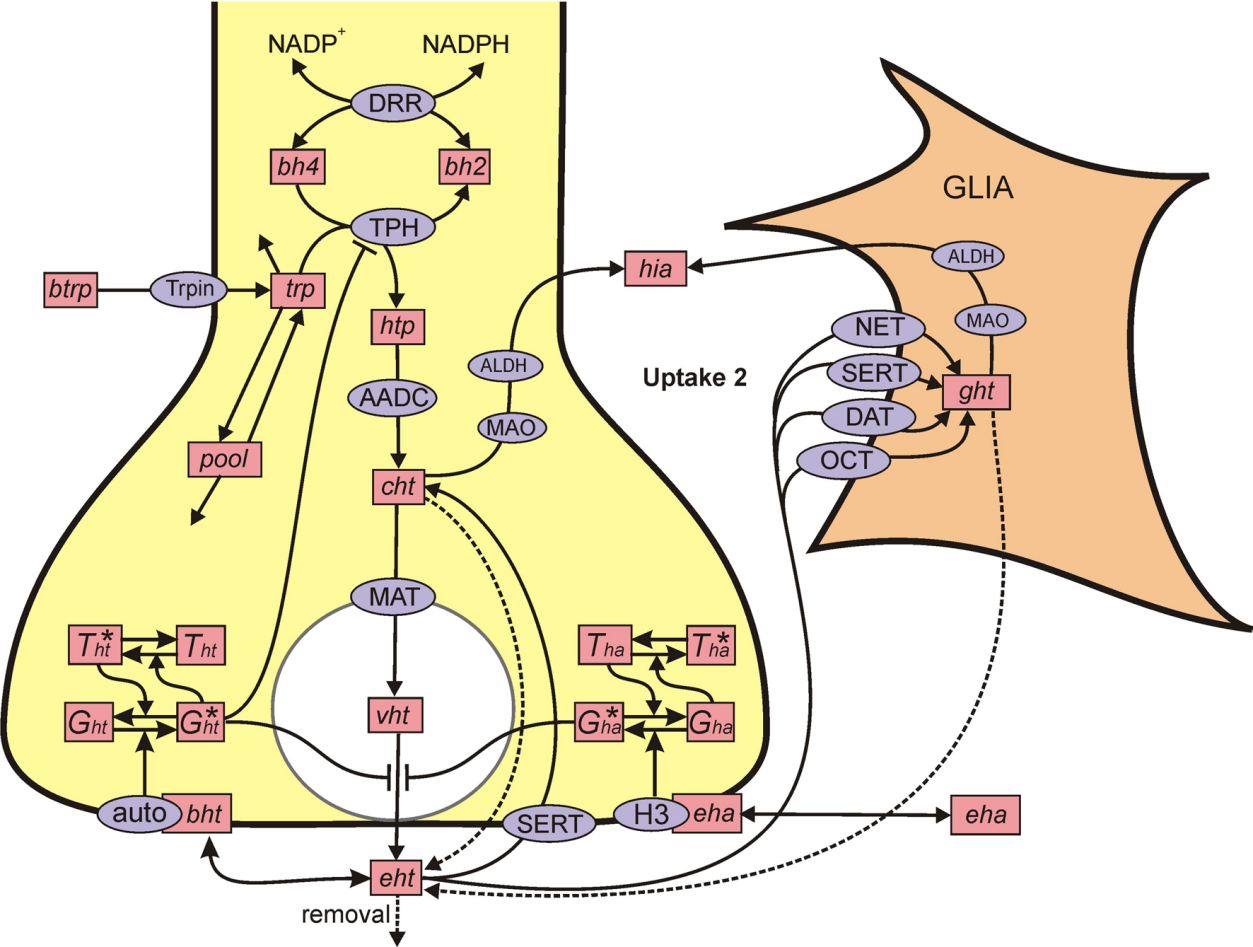
Schematic diagram of the model. The figure shows the reactions in the model. The rectangular boxes indicate substrates and blue ellipses contain the acronyms of enzymes or transporters. Full names of the substrates are given in Table 1. Names of enzymes and transporters are as follows: Trpin, neutral amino acid transporter; DRR, dihydrobiopterin reductase; TPH, tryptophan hydroxylase; AADC, aromatic amino acid decarboxylase; MAT, vesicular monoamine transporter; SERT, 5-HT reuptake transporter; auto, 5-HT autoreceptors; MAO, monoamine oxidase; ALDH, aldehyde dehydrogenase; NET, norepinepherine transporter; DAT, dopamine transporter; OCT, organic cation transporter. Removal means uptake by capillaries or diffusion out of the system. The figure was created by H. F. Nijhout for this study

These are the major changes to the model, but there are many minor changes too. A schematic diagram of the model is given in Fig. 1. The pink boxes contain the acronyms of the substrates (full names are in Table 1). A complete mathematical description of the model is given in “Methods” section where we discuss in detail the major changes. The parts of the model that are similar to the 2010 model are not discussed in detail; for those parts, we refer the reader to the the 2010 paper [1] for motivation and discussion.

All individuals, whether mouse or human, are different, and the variation is important for understanding experimental results and for precision medicine. This variability is self-evident in the experimental response curves that we discuss in “Variability in response to stimulation” section. The variability comes from several sources. First, it is known that the expression levels of most enzymes vary by about 25% or more between individuals [14–16]. This means that the $V_{max}$ values of the enzymes and transporters in the model must be assumed to vary by at least 25%. Second, Fig. 1 shows a diagram of our model of serotonin dynamics in a varicosity, but the parameters for that varocisity may depend on which projection region we are looking at. Indeed, tryptophan hydroxylase (TPH) expression varies considerably between different brain regions [17], as does SERT density [18], monoamine transporter (MAT) density [19], autoreceptor density [20, 21], and monoamine oxidase (MAO) density [22]. What this means is that the deterministic model described in the Methods and indicated schematically in Fig. 1 should not be regarded as “the” model of serotonin dynamics with “correct” parameters. Rather, it is a structure, which has the major players and interactions with reasonable kinetics, that can be used to investigate and interpret experimental data and to test hypotheses.

To incorporate and utilize the biological variation we have just described, we use our deterministic model of serotonin dynamics to create systems populations models. We choose new $V_{max}$ values for each of the enzymes and transporters in Fig. 1 by selecting independently from a uniform distribution between 75% and 125% of the normal value. We then run the model to equilibrium and record all the parameters, concentrations, and velocities at steady state. That is one virtual individual. If we do this 1000 times, we then have a database of 1000 individuals, each different from the other, and can perform the usual types of statistical analyses on the population to look for interesting aspects of population behavior. We can also vary selected subsets of the $V_{max}$ values to target specific questions and relationships. We use both the deterministic model and the systems population model throughout the Results.

In “The steady state” section, we discuss the steady state of the model and the steady state distribution of extracellular serotonin (*eht*) under different hypotheses on the autoreceptors. In “The effect of tryptophan input” section, we show the effect of tryptophan input on the steady state and in “The effect of meals” section the effect of meals. We use the deterministic model in “The dynamics of the autoreceptors” section to interpret experimental data on male and female mice and to illustrate the short time dynamics of the autoreceptors. In “Two serotonin uptake mechanisms and the effect of histamine” section, we use the deterministic model to understand data that show there are two uptake mechanisms and that the autoreceptors have long-lasting influence. We also show how histamine affects serotonin dynamics. In “Variability in response to stimulation” section, we examine data that shows variable response curves in populations of mice and ask how much variation in parameters in the model is necessary to produce the observed variation in the data. Finally, in “Using population models to understand drug efficacy and clinical measurements” section, we show how the systems population model can potentially be used to investigate specific biological and clinical questions.

## Methods

The mathematical model consists of 16 differential equations for the variables whose full names are listed in Table 1.

**Table 1:** Names of variables

In equations and text	Full name
*bh*2	Dihydrobiopterin
*bh*4	Tetrahydrobiopterin
*trp*	Tryptophan
*btrp*	Blood tryptophan
*htp*	5-Hydroxytryptamine
*cht*	Cytosolic serotonin
*vht*	Vesicular serotonin
*eht*	Extracellular serotonin
*hiaa*	5-Hydroxyindoleacetic acid
*pool*	The tryptophan pool
$G_{ht}^*$	Serotonin activated G-protein
$T_{ht}^*$	Serotonin activated T regulatory protein
$B_{ht}$	Serotonin autoreceptors bound to eht
*ght*	Glial serotonin
$G_{ha}^*$	Histamine activated G-protein
$T_{ha}^*$	Histamine activated T regulatory protein
$B_{ha}$	Histamine autoreceptors bound to eha
*eha*	Extracellular histamine

In the differential equations, reaction velocities or transport velocities begin with a capital V followed by the name of the enzyme, the transporter, or the process as a subscript. For example, $V_{\mathrm{TPH}}(trp,bh4,G_{ht}^*)$ is the velocity of the tryptophan hydroxylase reaction and it depends on the concentrations of its substrates, *trp* and *bh*4, as well as the activated G-protein, $G^*_{ht}$, via the autoreceptors.

$$\begin{aligned} \frac{d[bh2]}{dt} =  V_{\mathrm{TPH}}(trp,bh4,G_{ht}^*) - V_{\mathrm{DRR}}(bh2,\text{ NADPH },bh4,\text{ NADP}) \\ \frac{d[bh4]}{dt} =  V_{\mathrm{DRR}}(bh2,\text{ NADPH },bh4,\text{ NADP}) - V_{\mathrm{TPH}}(trp,bh4,G_{ht}^*)\\ \frac{d[trp]}{dt} =  V_{trpin}(btrp) - V_{\mathrm{TPH}}(trp,bh4,G_{ht}^*) - V_{\mathrm{pool}}(trp,pool) - k^{catab}_{trp}\cdot trp\\ \frac{d[htp]}{dt} =  V_{\mathrm{TPH}}(trp,bh4,G_{ht}^*) - V_{\mathrm{AADC}}(htp)\\ \frac{d[cht]}{dt} =  V_{\mathrm{AADC}}(htp) - V_{\mathrm{MAT}}(cht,vht) + V_{\mathrm{SERT}}(eht) -V_{\mathrm{CATAB}}(cht) - k_{cht}^{leak}\cdot cht\\ \frac{d[vht]}{dt} =  V_{\mathrm{MAT}}(cht,vht) - inhib_{ht}(G_{ht}^*)\cdot inhib_{ha}(G_{ha}^*) \cdot fire(t)\cdot vht \\ \frac{d[eht]}{dt} & =  inhib_{ht}(G_{ht}^*)\cdot inhib_{ha}(G_{ha}^*) \cdot fire(t)\cdot vht - V_{\mathrm{SERT}}(eht) - V_{\mathrm{U2}}(eht) - k_{eht}^{rem}\cdot eht + k_{ght}^{leak}\cdot ght \\ & \quad k_{cht}^{leak}\cdot cht - k_{5} \cdot eht \cdot (B_{ht}^{tot} - B_{ht}) + k_{6 }\cdot B_{ht}\\ \frac{d[hiaa]}{dt} =  V_{\mathrm{CATAB}}(cht) + V_{\mathrm{CATAB}}(ght) - k^{catab}_{hiaa}\cdot hia\\ \frac{d[pool]}{dt} =  V_{\mathrm{{pool}}}(trp,pool) - k^{catab}_{pool}\cdot pool\\ \frac{d[G_{ht}^*]}{dt} =  \beta _1 \left[ k_{1}\cdot B_{ht}^2\cdot (G_{ht}^{tot} - G_{ht}^*) - k_{2} \cdot T_{ht}^*G_{ht}^* \right] \\ \frac{d[T_{ht}^*]}{dt} =  \beta _2 \left[ k_{3} \cdot (G_{ht}^*)^2 \cdot (T_{ht}^{tot} - T_{ht}^*) - k_{4} \cdot T_{ht}^* \right] \\ \frac{d[B_{ht}]}{dt} =  \beta _3 \left[ k_{5} \cdot eht \cdot (B_{ht}^{tot} - B_{ht}) - k_{6} \cdot B_{ht} \right] \\ \frac{d[ght]}{dt} =  V_{\mathrm{U2}}(eht) - V_{\mathrm{CATAB}}(ght) - k_{ght}^{leak} \cdot ght\\ \frac{d[G_{ha}^*]}{dt} =  k_{7}\cdot B_{ha}^2\cdot (G_{ha}^{tot} - G_{ha}^*) - k_{8} \cdot T_{ha}^*G_{ha}^*\\ \frac{d[T_{ha}^*]}{dt} =  k_{9} \cdot (G_{ha}^*)^2 \cdot (T_{ha}^{tot} - T_{ha}^*) - k_{10} \cdot T_{ha}^*\\ \frac{d[B_{ha}]}{dt} =  k_{11} \cdot eha \cdot (B_{ht}^{tot} - B_{ha}) - k_{12} \cdot B_{ha}\\ \end{aligned}$$

Although *btrp* and *eha* are listed as variables, there are no differential equations for them because, in this paper, they are constant or their time courses are specified in the computational experiments. Table 2 gives the parameter choices and references for reactions that have standard Michaelis-Menten kinetics, single substrate, double substrate, or reversible. For reactions with non-standard kinetics, the serotonin autoreceptor, the $H_3$ receptor, and other parts of the model, detailed explanations follow.

**Table 2:** Kinetic parameters ($\mu$M,$\mu$M/h,/h)

Velocity	Parameter	Model value	Literature value	References
$V_{\mathrm{AADC}}$	Aromatic amino acid decarboxylase			
	$K_m$	160	160	[23]
	$V_{max}$	400		*
$V_{\mathrm{CATAB}}$	Catabolism of serotonin			
	$K_m$	95	94–95	[24, 25]
	$V_{max}$	4000		*
$V_{\mathrm{DRR}}$	Dihydropteridine reductase			
	$K_{bh2}$	100	4–754	[26, 27]
	$K_{\mathrm{NADPH}}$	75	29–770	[28–30]
	$V_{max}^f$	5000		*
	$K_{bh4}$	10	1.1–17	[29, 31]
	$K_{\mathrm{NADP}}$	75	29–770	[28–30]
	${V_{max}}^b$	3		*
$V_{\mathrm{MAT}}$	Vesicular monoamine transporter			
	$K_m$	.2	.123–.253	[32, 33]
	$V_{max}$	1230		*
$V_{\mathrm{POOL}}$	Linear exchange between *trp* and *pool*			
	$$ k_{topool}$$	9		*
	$$k_{frompool} $$	0.6		*
$V_{\mathrm{SERT}}$	Serotonin transporter			
	$K_m$	.06	0.05–1	[34]
	$V_{max}$	250		*
$V_{\mathrm{TPH}}$	Tryptophan hydroxylase			
	$K_{trp}$	40	40	[35]
	$$ K_{bh4}$$	20	20	[35]
	$$V_{max} $$	278		*
	$K_i$ (substrate inhibition)	1000	970	[35]
$V_{trpin}$	Neutral amino acid transporter			
	$K_m$	330	64	[36]
	$$ V_{max}$$	700		*
$$V_{\mathrm{U2}}$$				
	$K_m$	.17	0.17	[37, 38]
	$V_{max}$	14		[37, 38]
Linear diffusion or catabolism				
	$k^{catab}_{trp}$	2		*
	$k^{leak}_{cht}$	1		*
	$k_{ght}^{leak}$	1		*
	$k^{catab}_{hiaa}$	1	.82	[39]
	$k^{catab}_{pool}$	1		*
	$k_{eht}^{rem}$	40		*
The serotonin autoreceptor				
	$G_{ht}^{tot}$	10		*
	$T_{ht}^{tot}$	10		*
	$B_{ht}^{tot}$	10		*
	$k_1$	20		*
	$k_2$	200		*
	$k_3$	30		*
	$k_4$	200		*
	$k_5$	36000		*
	$k_6$	20000		*
	$\beta _1$	1		*
	$\beta _2$	1		*
	$\beta _3$	1		*
The histamine $H_3$ receptor				
	$G_{ha}^{tot}$	1		*
	$T_{ht}^{tot}$	60		*
	$B_{ht}^{tot}$	10		*
	$k_{7}$	4.32		*
	$k_{8}$	1.296		*
	$$ k_{9}$$	14.4		*
	$$ k_{10}$$	25.92		*
	$$ k_{11}$$	432		*
	$$ k_{12}$$	1440		*
Other constants (varied in some experiments)				
	NADP	26		*
	NADPH	330		*
	*btrp*	96		*
	*eha*	1.39		*

*See text

*Tryptophan hydroxylase (TPH)* The kinetics of TPH show substrate inhibition. The Michaelis constants and the substrate inhibition constants are given in Table 3 with references. In [1], the inhibition of TPH by the serotonin autoreceptor was modeled by having the current concentration of *eht* affect synthesis. However, in this paper we have a kinetic model of the autoreceptor (see below), so TPH is inhibited or excited by whether the concentration of the G-protein, $$G^*_{ht}$$, is above or below its equilibrium value, $$ G^*_{hteq}$$. Little is known about the kinetics of this effect, so we take the slope of this effect to be 2.5, that is, $$inhibsyn(G^*_{ht}) = 1 - (2.5)(G^*_{ht} - G^*_{hteq}).$$ The full kinetics of TPH are:

$$\begin{aligned} V_{\mathrm{TPH}}(trp,bh4,G^*_{ht}) \; = \; \frac{V_{max}(trp)(bh4)}{(K_{trp} + (trp) + \frac{(trp)^2}{K_i})(K_{bh4}+(bh4))}\cdot inhibsyn(G^*_{ht}). \end{aligned}$$

*Uptake 2 (*$V_{\text{ UP2 }}$). It is not a new idea that serotonin may be taken up from the extracellular space not only by SERTs but also by other transporters. Shaskan and colleagues suggested this in the 1970s [11] and recently the therapeutic potential for depression of this second uptake has been emphasized by Daws [12] and Horton [13]. The hypothesis is that serotonin can be taken up into glial cells by the dopamine transporter (DAT), the norepinepherine transporter (NET), and the organic cation transporter (OCT) as well as the SERTs. We refer to this uptake into glial cells collectively as Uptake 2 and it is depicted schematically in Fig. 1. The presence of two uptake mechanisms was confirmed by the in vivo experiments of Hashemi [7], one high affinity but low capacity (SERTs) and one low affinity and high capacity (Uptake 2). In the microdialysis experiments of Bunin and Wightman [37, 40] on tissue from the dorsal raphe (DR) and the substantia nigra pars reticulata (SNr), large quantities of serotonin were released into the extracellular space and the concentration of serotonin in the effluent was measured over time. The almost linear decline of serotonin over short times allowed them to estimate the $V_{max}$ of the uptake and the later decline allowed them to estimate the $K_m$. The $K_m$ in both regions was 170 nM and the $V_{max}$ varied from 1800 nM/s in the DR to 780 nM/s in the SNr. These values are consistent with fast responses and the fast part of the decline in the hybrid responses measured in vivo by Hashemi [7], so we believe that Bunin and Wightman were, in fact, measuring Uptake 2 and not the SERTs. Thus we take the $K_m$ of Uptake 2 to be $.17 \upmu$M. Bunin and Wightman were measuring microdialysis effluent from tissue slices. The Hashemi Lab measures the time dynamics in the extracellular space with microelectrodes in mice in vivo (see below). We expect that the $V_{max}$ will vary enormously depending on how many glial cells are near the electrode. So we take our minmal baseline value to be $V_{max} = 14\,\mu$M/hr but expect that the $V_{max}$ will be much higher in some circumstances.

The hybrid responses (“Two serotonin uptake mechanisms and the effect of histamine” section) show that Uptake 2 only operates above some threshold that is (usually) above the basal level of *eht*, which in our model is 60 nM. This is consistent with the idea that Uptake 2 is low affinity. We accomplish this in the model by multiplying the kinetics of Uptake 2 by a factor *H* that equals zero below 60.5 nM, increases linearly to 1 at 80.5 nM and is 1 above that (the factor 1000 is because *eht* is measured in nM).

$$\begin{aligned} V_{\mathrm{U2}}(eht) \; = \; H(1000\cdot eht) \frac{V_{max}\cdot (eht)}{K_m + eht}. \end{aligned}$$

*The* $5HT_{1B}$*autoreceptor* As described in the Introduction, the experiments in the Hashemi Lab showed clearly that autoreceptor effects are long-lasting (seconds to minutes) and that they persist even when the *eht* concentration returns to normal. It is this persistence that drives the *eht* concentration well below baseline in the majority of stimulation experiments. Almost certainly these delays are because the cellular machinery by which the autoreceptors act takes time to turn on and turn off. In our original model [1], the autoreceptors act instantaneously because the inhibition of release when *eht* rises depended on the current value of *eht*. The experimental advances require a more sophisticated model of the action of autoreceptors.

The $5HT_{1B}$ autoreceptor is in the family of G protein-coupled receptors (GPRC) [41], and there is a large literature on the structure and modeling of GPRCs including diverse second messengers, geometrical configuration, and possible dimerization [42–44]. The binding of an *eht* molecule to the $5HT_{1B}$ autoreceptor causes the release of a G-protein subunit that stimulates a signaling cascade that results in inhibition of release and synthesis. Our purpose here is to create a simple model for our autoreceptor dynamics. In our model, $G_{ht}$ represents $G_{\alpha }-GDP$ (the inactive G-protein subunit) and $G_{ht}^*$ represents $G_{\alpha }-GTP$ (the signaling G-protein unit). Most G-protein signals are limited by RGS molecules (regulators of G-protein signaling) that stimulate the G-protein subunit to rebind [45]. $T_{ht}$ represents the inactive RGS protein and $T_{ht}^*$ represents the active RGS protein. A schematic diagram of this chemistry is in Fig. 1 and the details are in the differential equations for $G_{ht}^*, T_{ht}^*$ and $B_{ht}$. The bound *eht* stimulates the conversion of $G_{ht}$ to its active form $G_{ht}^*$ , and $G_{ht}^*$ stimulates the conversion of $T_{ht}$ to its active form, $T_{ht}^*$. In turn, $T_{ht}^*$ stimulates the deactivation of $G_{ht}^*$. We assume that total autoreceptors, total G protein, and total RGS protein are constants.

It has been understood since the 1970s that the $5HT_{1B}$ autoreceptors sense *eht*. When *eht* goes up, they inhibit both the synthesis of serotonin and the release of serotonin from the vesicles and when *eht* goes down they facilitate synthesis and release [46–51]. Thus *eht* provides a kind of end-point feedback for the entire serotonergic system from tryptophan in the plasma to *eht* in the extracellular space. Unfortunately, relatively little is known about the amplitude of these effects or the ranges over which they operate. Furthermore, these kinetics likely vary from varicosity to varicosity and cell to cell depending on the expression level of the $5HT_{1B}$ autoreceptors. We take relatively simple formulas for the effect of $G^*_{ht}$ on release and synthesis:

$$\begin{aligned} inhib(G_{ht}^*) = 1.89 - (s)(G_{ht}^*-G_{hteq}^*),  inhibsyn(G_{ht}^*) = 1 - (s)(G_{ht}^*-G_{hteq}^*), \end{aligned}$$

where $G_{hteq}^*$ is the equilibrium value of the activated G-protein and *s* is the slope of the effect, higher *s*, stronger effect. Our normal values for *s* are $s = 12.5$ for the inhibition of release and $s = 2.5$ for inhibition of synthesis. As we will see below, the experiments in the Hashemi Lab give some information on the strength *s* of the autoreceptor effect. It is also known [52] that the autoreceptors modulate reuptake, but this effect is not included in the model. Finally, we remark that serotonin is known to be an appetite suppressant [53], and therefore the concentration in the extracellular space *should* be at least somewhat sensitive to meals.

*The histamine* $H_3$*receptor.* We’ve taken the model for the $H_3$ receptor from our paper [54]. The schematic diagram is in Fig. 1 and the differential equations for $B_{ha}$, $G^*_{ha}$, and $T^*_{ha}$ are given above and the values of the parameters are in Table 2. $G^*_{ha}$ inhibits the release of serotonin by multiplying release by the function $inhib_{ha}(G^*_{ht}) = 1 - (5)(G^*_{ha} - G^*_{haeq}),$ where $G^*_{haeq}$ is the equilibrium value of the activated G-protein. Note that, at equilibrium, this multiplier is = 1.

*Serotonin binding protein and release* In our model there is a constant basal rate of serotonin release at steady state. In the experiments on mice in the Hashemi Lab, the medial forebrain bundle (MFB) is stimulated for 2 s. The antidromic spikes propagate back to stimulate the serotonin neurons in the dorsal raphe nucleus (DR) which in turn send spikes to projection regions in which extra serotonin is released. The FSCV probe in the projection region (see below) measures the concentration of serotonin in the extracellular space over 30 s. The question is how should we model the release of serotonin over the 30 s period—the first term in the differential equation for *eht*? The question is complicated by the existence of serotonin binding protein (SBP) that is attached to the inner wall of vesicles and binds serotonin tightly [55, 56]. We assume that the dissociation is a first order reaction

$$\begin{aligned} SBP-serotonin \; \overset{b}{\longrightarrow } \; SBP \; + \; serotonin. \end{aligned}$$

If we start with one unit (nM) of SBP-serotonin being dumped into the extracellular space at time zero, then $SBP(t) = e^{-bt}$ and $serotonin(t) = 1 - e^{-bt}$. The rate of release of serotonin is the derivative, $be^{-bt}.$ However, we are stimulating for two seconds, so SBP-serotonin complexes are continuously dumped into the extracellular space between $t=0$ and $t=2$ s. Assume that the rate of dumping is 1 nM/s, so in 2 s, 2 nM of the complex are dumped. What is the rate of appearance, *R*(*t*), of serotonin for $t \le 2$ and $t > 2?$

$$\begin{aligned} R(t) \; = \; \int _0^{t} \chi _{[s,2]}be^{-b(t-s)} \, ds  \text{ for } \;\;\; t \le 2, \end{aligned}$$

and

$$\begin{aligned} R(t) \; = \; \int _0^{2} \chi _{[s,2]}be^{-b(t-s)} \, ds  \text{ for } \;\;\; t > 2. \end{aligned}$$

Here $\chi _{[s,2]}$ is the function that is 1 on the interval [*s*, 2] and zero otherwise. A straightforward calculation shows that:

$$\begin{aligned} R(t) = {\left\{ \begin{array}{ll} 1 - e^{-bt} &{} \text { if } t \le 2,\\ e^{-b(t-2)} - e^{-bt} &{} \text { if } t > 2. \end{array}\right. } \end{aligned}$$

Thus, for a 2 s stimulation, as was the case for the data used below, the rate of release will be proportional to $fire(t) = \text{ basal } \text{ rate } \;+\; r\cdot R(t)$ where *r* is the strength of the stimulation. Unfortunately, the dissociation constant *b* (inverse seconds) is not known, but we think it is in the range $0.5 \le b \le 2$ from our simulations of the Hashemi data, so we’ll take $b=1$ as our baseline case. The release of serotonin into the extracellular space will also be proportional to *vht* and it will also depend on the inhibition from the serotonin autoreceptors and the histamine $H_3$ receptor. Thus, overall release as a function of time will be

$$\begin{aligned} inhib_{ht}(G_{ht}^*)\cdot inhib_{ha}(G_{ha}^*) \cdot fire(t)\cdot vht , \end{aligned}$$

which is the first term in the differential equation for *eht* above.

*Minor changes* Since we have added a glial cell compartment, serotonin is catabolized in the neuron and in the glial cell, and not in the extracellular space (as in the 2010 model). We have added leakage of serotonin from the cytosol to the extracellular space [57], indicated by the dashed lines in Fig. 1. Because most of *vht* is bound to serotonin binding protein, we have reduced the linear back diffusion from the vesicles to the cytosol from 40 to 1 (contained in the formula for $V_{\mathrm{{MAT}}}$). The linear removal of *eht* from the extracellular space represents diffusion out of the tissue and uptake by the circulatory system.

*A systems population model* All individuals, whether mouse or human, are different, and the variation is important for understanding experimental results and for precision medicine. We investigate this biological variation by creating a population model of the deterministic model given above. It is known that the expression levels of most enzymes can vary by about 25% or more between individuals [14–16]. Therefore, to create a systems population model, we choose new $V_{max}$ values for each (or a subset) of the enzymes and transporters in Fig. 1 by selecting independently from a uniform distribution between 75% and 125% of the normal value. In some cases we choose larger variation (“Using population models to understand drug efficacy and clinical measurements” section). We then run the model to steady state and record all the concentrations and velocities. That is one virtual person (or mouse). If we do this 1000 times, we obtain a database of virtual individuals that we can analyze using the usual statistical tools. The difference is that all of these individuals have the same set of differential equations; only the coefficients are different. So we can experiment with the model to find the mechanistic reasons for particular statistical phenomena (as we will do below). And, we can sometimes verify the results of our populations models by comparing to known databases (for example, see the systems population model for one-carbon metabolism in [58]).

*Caveats* Every mathematical model model is an oversimplified representation of complicated and variable physiology. In this model we chose $K_m$ values for enzymes and transporters from the literature but chose $V_{max}$ values so the model would reproduce steady state values consistent with the literature. In any case, $V_{max}$ values depend on enzyme expression levels that vary in time and differ widely from person to person [14–16]. We have included a glial cell in the model to represent all the non-neuronal cells that can take up and catabolize serotonin. Good measurements of the size of the vesicular compartment and the size of the extracellular space (per varicosity) are not available and so we treat those compartments as though they are the same size. We have referred to the “concentration” of autoreceptors and bound autoreceptors but we have no good way of estimating effective concentrations for the autoreceptors that are on the varicosity surface, so the units are arbitrary.

*Using the model to make predictions* In the Background, we explained that one should expect as much as 25% variation in many of the parameters of the model because of biological variation in individual cells or because one is studying different brain regions. Thus our “standard” model with the parameters from Table 2 should be regarded as a model for an “average” mouse in an average brain region. We expect large individual variation in individual mice and, indeed, that is what one sees in the data and in our systems population model (Fig. 6a, b). Given this variation, what does it mean to claim that we have constructed a “good” model. Certainly, it does not mean that our standard deterministic model with a fixed set of parameters is “right” and explains everything. We believe that: (1) A good model should contain the major players that biologists would say are involved in the phenomena we are studying, and the model should be constructed using as much as possible experimental information on concentrations and kinetics; (2) One should be able to test hypothesis by running simulations of the model; (3) The model should be useful to provide a theoretical framework to help experimentalists interpret data.

We use the model in several different ways. Often we determine the parameter changes that are necessary so that the model curves or model data points fit the experimental ones (as in Results 3.4 and 3.5). These parameter changes (such as the strength of Uptake 2 or the strength of the $5HT_{1B}$ autoreceptors or the rapidity and duration of the autoreceptor response) are then predictions that could be verified experimentally. Sometimes we conduct theoretical experiments and don’t fit data as in “The effect of meals” section where we ask how much variation in *eht* should one expect from the daily variation in tryptophan input due to meals. Even in such cases, the theoretical results are predictions that could be verified experimentally. Finally, sometimes we ask interesting theoretical questions of the model such as how much variation in model parameters is necessary to obtain the variation seen in the data (“The steady state” and “Variability in response to stimulation” sections). Experimental validation or invalidation of model predictions will suggest improvements to the model and better theoretical understanding. Indeed, this new model was necessary because of the inability of the older model [1] to explain the long-lasting autoreceptor effect seen in experimental data (“The dynamics of the autoreceptors” and “Two serotonin uptake mechanisms and the effect of histamine” sections). Thus, we don’t expect “the model” to be fixed, but to evolve in response to new experiments.

*Fast scan cyclic voltammetry* To make carbon fiber microelectrodes (CFM) for fast scan cyclic voltammetry (FSCV), a 7 $\upmu$m carbon fiber (Goodfellow Corporation, PA, USA) was aspirated through a borosilicate glass capillary (0.6 mm external diameter, 0.4 mm internal diameter, AM systems, Inc., Sequim, WA). This capillary was pulled to a fine tip via a micropipette puller (Narishige Group, Tokyo, Japan) to form a seal around the carbon. Silver conducting epoxy paint was used to make electrical connection between the carbon fiber and hardware connections. The exposed carbon fiber was cut to $150 \upmu \hbox {m}$ and Nafion was electropolymerized onto the electrode surface for improved selectivity [59 original MS]. To make FSCV measurements, the CFM was directly implanted into the brain region of interest (coordinates given below). The serotonin-selective potential waveform, the Jackson waveform described in detail elsewhere [59] (0.1 – 1.0 to -0.2 – 1.0 V at 1000 Vs-1) was applied to the carbon at 10 Hz. In the time between waveform application, the potential was held at the resting potential of 0.1 V to minimize interfering species and to preconcentrate the analyte of interest and increase sensitivity. At discrete potentials, the analyte of interest becomes oxidized (on the anodic scan) and reduced (on the cathodic scan). Oxidation and reduction events generate Faradaic currents, which can be observed by subtracting out the large, capacitative background current. As a result, FSCV is used for measuring evoked changes in nanomolar but cannot measure absolute concentrations. Data from FSCV is analyzed via cyclic voltammograms (CVs), which is the plot of current as a function of potential applied, and current vs. time (iT) curves. The former provides qualitative information about the identity of the species while the latter provides quantitative information about release and reuptake of the analyte in question via comparison of the signal to calibration curves constructed with standard concentrations.

*Animals and surgical procedures* All protocols described herein are in accordance with the Guide for the Care and Use of Laboratory Animals at the University of South Carolina, and have been approved by the Institutional Animal Care and Use Committee (IACUC) at this Institution, which operates with accreditation from the Association for Assessment and Accreditation of Laboratory Animal Care (AAALAC).” Anesthesia is induced via intraperitoneal injections of urethane (25% dissolved in 0.9% NaCl solution, Hospira, Lake Forest, IL, USA). Once the animals were fully anesthetized as assessed with lack of toe pinch reflex, they were secured into a stereotaxic instrument (David Kopf Instruments, Tujunga, CA, USA) and surgery was performed to implant the electrodes into appropriate coordinates:

Posterior Hypothalamus: (CFM: AP -2.45, ML: +0.50, DV: -5.45 to -5.55 w.r.t. Bregma; stimulating electrode: AP: -1.07, ML: +1.10, DV: -5.00 w.r.t Bregma).

Hippocampus: (CFM: AP -2.91, ML: +3.35, DV: -2.5 to -3.0 w.r.t. Bregma; stimulating electrode: AP: -1.58, ML: +1.0, DV: -4.8 to -5.0 w.r.t. Bregma).

Substantia nigra pars reticulata: (CFM: AP: -3.28, ML: +1.4, DV: -4.2 to -4.8 w.r.t. Bregma; stimulating electrode: AP: -1.58. ML: +1.0, DV: -4.8 to -5.0 w.r.t. Bregma).

A pseudo-Ag/AgCl electrode was placed in the contralateral hemisphere to complete the circuit and act as a reference electrode. A heating pad was used to maintain body temperature around 37 $^\circ$C throughout the procedures and experiments (Braintree Scientific, Braintree, MA, USA). We reported in prior work that a power analysis recommended a minimum n size of 3.5 (rounded to 4) and maintained that there, along with a strict animal exclusion criteria (i.e. animals that did not survive the experiment, animals in which the electrodes broke and animals in which the identity of the analyte could not be confirmed electrochemically via inspection of cyclic voltammograms [60].

*Numerical computations* The differential equations in Table 1 were solved using the ODE15s in MatLab. The parameters were as indicated in Table 2, except as indicated explicitly in the discussion and figures about each simulation experiment. The MatLab code is available from the authors.

## Results and discussion

### The steady state

Table 3 shows the concentrations of the variables at the normal steady state of the model and also shows many of the velocities. Concentrations are in $\upmu$M except for *eht* which is in nM, and velocities are in $\upmu$M/h. A major change from our 2010 model is that the steady state *eht* concentration is now 60 nM instead of 0.7 nM. The Hashemi Lab has repeatedly verified that the extracellular serotonin concentration in mice is in the range 40–80 nM [60–62], although it varies considerably between the different regions to which the dorsal raphe and medial raphe project. The steady state concentration of histamine in the extracellular space is taken from [54]. We are assuming that the cutoff for Uptake 2 (see “Methods” section) is normally at 60.5 nM, which is why there is no serotonin in the glia at the normal steady state. Uptake 2 comes into play during the stimulation experiments (below) when serotonin release is stimulated.

**Table 3:** The steady state

Variable	Concentration ($\upmu$M)	Velocity	Rate ($\upmu$M/h)
*bh*2	0.1	$V_{trpin}$	157.8
*bh*4	0.9	$V_{\mathrm{TPH}}$	3.99
*trp*	20.2	$V_{\mathrm{AADC}}$	3.99
*btrp*	96	$V_{\mathrm{CATAB}}$	1.58
*htp*	1.61	$V_{\mathrm{MAT}}$	127.4
*cht*	0.04	Release	127.4
*vht*	67.5	$V_{\mathrm{SERT}}$	125.1
*eht*	0.060	Removal	2.4
*hiaa*	1.59	$V_{\mathrm{U2}}$	0.0
*pool*	113		
$G_{ht}^*$	0.86		
$T_{ht}^*$	1.01		
$B_{ht}$	0.97		
*ght*	0.0		
$G_{ha}^*$	0.69		
$T_{ha}^*$	12.69		
$B_{ha}$	2.94		
*eha*	1.39		

The concentration of *btrp* is $96\upmu \hbox {M}$ as found by [36]. Note that only a small fraction of the *trp* imported from the blood goes to the synthesis of serotonin, the rest being catabolized or taken to *pool* that represents all of the other uses of *trp* in the cell. The synthesis pathway is quite slow compared to release, reuptake via the SERTs, and packaging into vesicles by MAT. At the normal steady state, approximately 98% of the released serotonin is returned to the cytosol by the SERTs; the other 2% (removal) diffuses out of the tissue or is taken up by blood vessels. The steady state concentration for *hiaa* is consistent with the range found in [63].

**Fig. 2:**
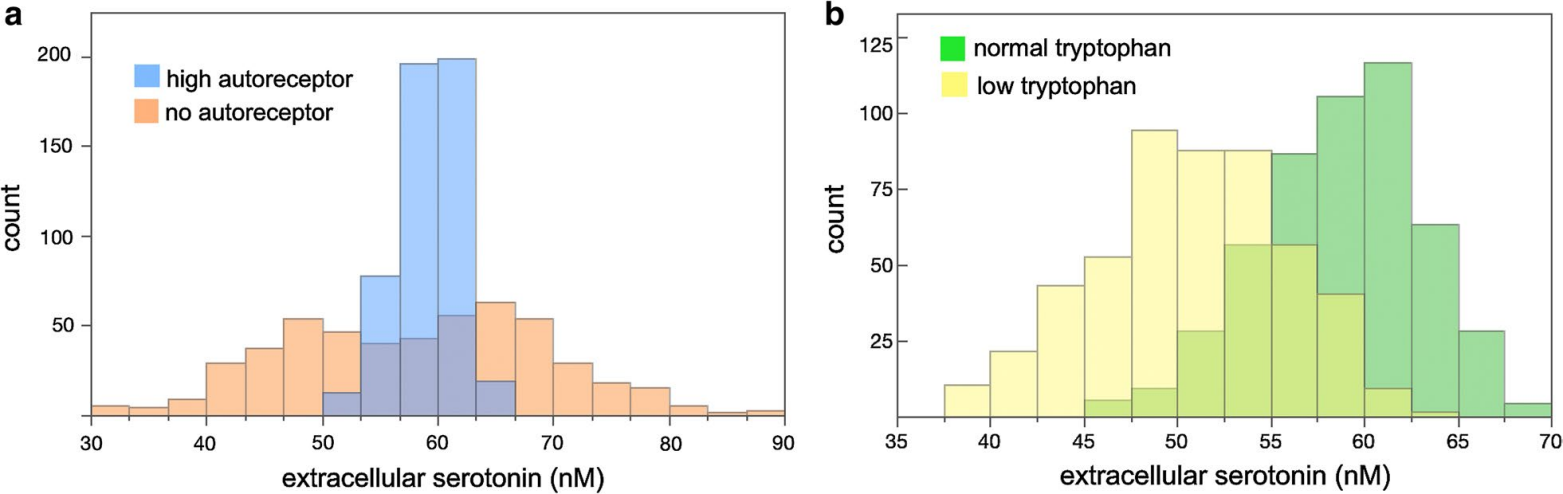
Autoreceptors effect the distribution of *eht* values in the systems population model. In our systems population model we varied the strength of *fire*(*t*) and the $V_{max}$ values of Trypin, TPH, AADC, MAT, MAO, Uptake 2, and SERT by 25%. **a** Shows the distribution of *eht* if there is no autoreceptor effect (pink bars) or if the autoreceptor effect is twice as strong as normal (blue bars). The green bars in **b** show the distribution of *eht* if the autoreceptor effect is “normal” (12.5 for the slope of *inhib* and 2.5 for the slope of *inhibsyn*). The green bars are similar to distributions measured in the Hashemi Lab (data not shown). The yellow bars show the distribution of *eht* if blood tryptophan is lowered from its normal value of $96\upmu \hbox {M}$ to $50\upmu \hbox {M}$. The distribution of *eht* moves substantially lower

The steady state shown for *eht* (60 nM) is for an “average” mouse (or an “average” person), but, of course, each individual is different and will have different parameters, a different steady state, and different responses to stimulation as we will see below. The natural variation in enzyme expression levels between individuals is approximately 25%, [14, 64, 65]. Our systems population model (see Methods) allows us to see how the variation affects the distribution of *eht* levels in the population. We allowed the $V_{max}$ values of TRPin, TPH, AADC, MAT, MAO, Uptake 2, and SERT to vary by 25% above and below their normal values independently. In addition we allowed *fire*(*t*) to vary 25% above and below its normal value and the slope of the function $inhib(G_{ht}^*)$ to vary by $25\%.$ We are particularly interested in the effect of the $5HT_{1B}$ autoreceptors, so we computed the *eht* distribution in three cases: standard autoreceptors, high autoreceptors (twice as strong as standard), and no autoreceptors. For standard autoreceptors, the distribution of *eht* is given by the green bars in Fig. 2b. The distribution is broad with a range of 45 to 70 nM, a mean of 58.7 nM and a standard deviation of 4.3 nM. This distribution is similar to the distributions seen in the pre-frontal cortex of mice in the Hashemi Lab [60]. For no autoreceptors, the distribution is given by the pink bars in Fig. 2a. It is much broader, with a range of 30 to 90 nM, a mean of 58.2 nM and standard deviation of 11.1 nM. For high autoreceptors, the resulting quite narrow distribution of *eht* values is shown by the blue bars in Panel A. The distribution has a range of 50 to 67 nM, a mean of 59.2 nM, and a standard deviation of 2.7 nM. This shows the effect of the $5HT_{1B}$ autoreceptors on the distribution of *eht*. Using data on the distribution of *eht* values, we can compute the likely value of the slopes, s, of the functions *inhib* and *inhibsyn*, which is how we arrived at our “normal” values of 12.5 and 2.5, that is, we can estimate the strength of the autoreceptor effect. Note that all the distributions fall much more rapidly to the right of the peak than to the left. This is because Uptake 2 operates above the normal steady state and transports *eht* into the glia. It is likely that Uptake 2 is an evolutionary mechanism to prevent serotonin syndrome [66, 67].

Each time that we run the systems population model, we get slightly different distributions of *eht*. The distributions shown in Fig. 2 are typical examples of the three cases: standard autoreceptors, high autoreceptors, and no autoreceptors. The systems population model demonstrates the strong effect of the autoreceptors on the distribution of *eht* values in variable populations.

### The effect of tryptophan input

Many studies have shown that low brain serotonin is associated with depression [68–75], so there has been great interest in investigating whether and how brain serotonin can be affected by diet. For several reasons, it is not easy to estimate how changes in dietary tryptophan affect *eht*. Tryptophan, the amino acid precursor to serotonin, competes for the L-transporter at the blood-brain barrier with the other neutral amino acids [76–78], and since the L-transporter is normally operating at close to saturation the amount of blood tryptophan transported depends on the concentrations of other amino acids. Furthermore, much tryptophan in the blood is bound to albumin [79] and Fernstrom has shown that because of this binding the amount of tryptophan transported depends on the order of protein and carbohydrate consumption [80]. Finally, tryptophan hydroxylase, TPH, shows substrate inhibition, so if cytosolic tryptophan rises, the synthesis rate can go up or down depending on the concentration of tryptophan.

**Fig. 3:**
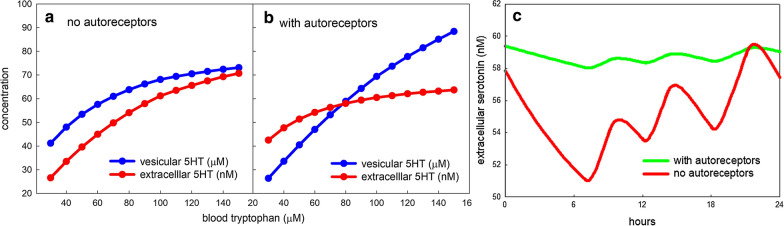
The effect of tryptophan input. **a** Shows the steady state values of vesicular serotonin, *vht* (blue curve), and extracellular serotonin, *eht* (red curve), over a range of values of blood tryptophan ($96\upmu \hbox {M}$ being normal) if the autoreceptors are turned off. **b** Shows the analogous curves if the autoreceptor effect is normal. The *vht* curve varies much more and the *eht* curve varies much less (for explanation, see the text). **c** Shows the effect of three daily meals on the extracellular concentration of *eht* with no autoreceptor effect (red curve) and normal autoreceptors (green curve). The autoreceptors greatly dampen the fluctuations of *eht* due to meals

Nevertheless, we can use the model to see how vesicular serotonin, *vht*, and extracellular serotonin, *eht*, change as plasma tryptophan (*btrp*) changes. Figure 3a shows that both *vht* (blue curve) and *eht* change substantially if the autoreceptor effect is turned off. Panel b shows that, when the autoreceptor effect is turned on *vht* changes dramatically but *eht* varies much less than in Panel a. This is because when *eht* is below normal, the autoreceptors increase release, which depletes *vht* and the opposite occurs when *eht* is above normal. The magnitude of the change of *vht* is consistent with experiments in the Hashemi Lab [81] where serotonin cells were incubated in media with $40\upmu \hbox {M}$ tryptophan and $140\upmu \hbox {M}$ tryptophan, respectively. On electrical stimulation, the high tryptophan cells released approximately twice as much *eht* as the low tryptophan cells. Since release is proportional to *vht*, this difference is what would be predicted by the model results in Fig. 3b.

We can also examine the effect of low tryptophan by using the systems population model. The slope of the $5HT_{1B}$ autoreceptor was varied by 25% around its standard value of $s = 12.5$, and similarly the 7 enzymes and *fire*(*t*) were varied by 25% as discussed in the caption to Fig. 2. The green bars in Fig. 2 show the distribution of *eht* if blood tryptophan is held constant at its normal value of $96 \upmu$M, while the yellow bars show the distribution of *eht* if blood tryptophan is held constant at $50 \upmu$M. As can be clearly seen, lowering blood tryptophan moves the *eht* distribution substantially to the left.

###  The effect of meals

During a 24 h period, the plasma amino acid concentration can vary by as much as a factor of 6 but more typically varies by a factor of 2 to 4 [36, 82, 83] because of meals. This means that *vht* and *eht* are never really at steady state but vary during the day and we can calculate the effect of this variation on the serotonin system. As we will see, this gives a good demonstration of the homeostatic effect of the autoreceptors and Uptake 2. To simulate meals, we assume that the normal concentration of blood tryptophan is doubled for two hours after breakfast and lunch, and for 3 h after dinner, and is correspondingly lower at other times so that the average concentration is 96 $\upmu$M as we assume at steady state. In our model, it is the activated G-coupled protein, $G^*_{ht}$, that inhibits release by multiplying the rate of release by the factor:

1 $$\begin{aligned} inhib(G^*_{ht}) =  1 - (s)(G^*_{ht} \; - \; G^*_{hteq}). \end{aligned}$$

where $s = 12.5$, normally. If $s=0$, there is no autoreceptor effect because *eht* and $G^*_{ht}$ don’t affect release. If $s = 12.5$, the normal value, the autoreceptors inhibit release when *eht* is above its normal value (and therefore $G^*_{ht}$ is above its normal value). And, when *eht* is below its normal value, $inhib(G^*_{ht})$ is above its normal value so release (per action potential) is increased. The dynamic changes in *eht* due to meals are shown in Fig. 3c for no autoreceptors (red curve) and normal autoreceptors (green curve). When there is no autoreceptor effect, *eht* oscillates between (approximately) 51nM and 60 nM during the day. With the normal autoreceptor effect, the oscillations of *eht* go from approximately 58 nM to 60 nM. The average *eht* in both cases is well below the steady state value of 60 nM. This is because upward deviations of *btrp* do not have much effect on *eht*, but downward deviations have a large effect (see Fig. 3b). The reason is that Uptake 2 is quite strong above 60 nM and it limits the upward deviations of *eht* when *btrp* goes up. This is the same reason that the distribution of normal *eht* steady states in the population model (the green bars in Fig. 2b) has a long tail to the left but falls off sharply to the right of 60 nM.

### The dynamics of the autoreceptors

**Fig. 4:**
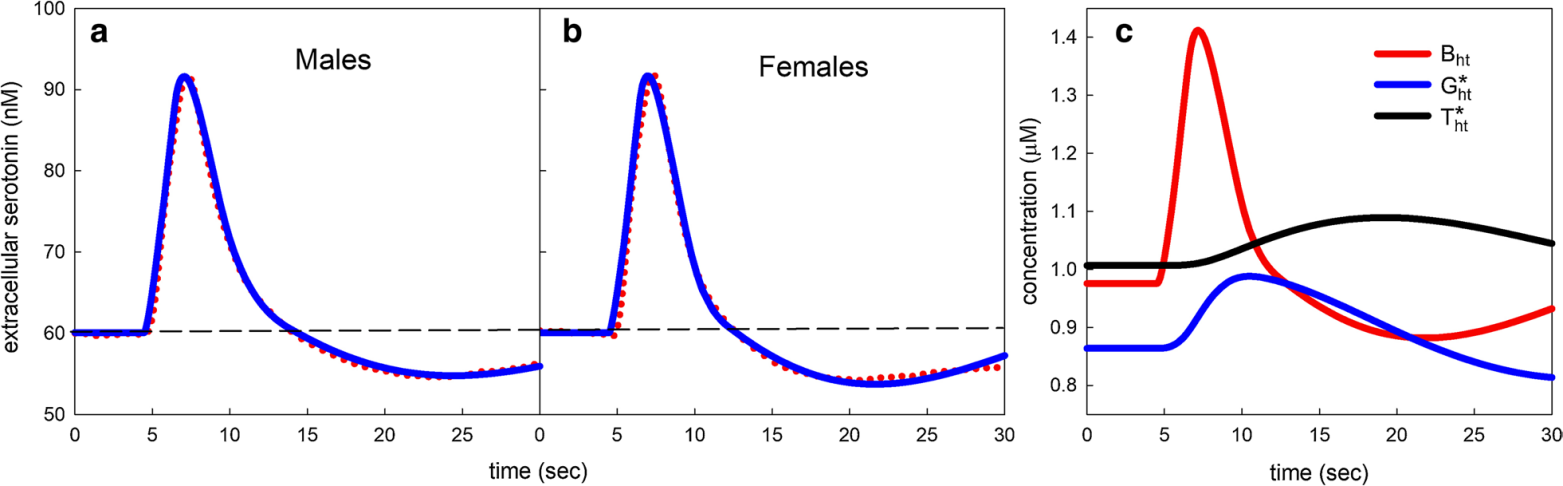
Male and female responses and autoreceptor dynamics. The red dots in **a**, **b** show the experimental averages of (n = 23) male and (n = 23) female responses of extracellular serotonin, *eht*. The blue curves show the model simulations. Parameters for the simulations are given in Table 4 and discussed below. The dashed horizontal line is the average baseline concentration of *eht* in the CA2 region of the hippocampus. The male and female average responses are similar. **c** Shows the dynamics of the three variables, $B_{ht}, G_{ht}^*, T_{ht}^*$ of the serotonin $5HT_{1B}$ autoreceptor dynamics. “See the Discussion in the text”. The experimental data is replotted from [62]

In the previous sections we illustrated the effects of the $5HT_{1B}$ autoreceptors on steady state values of *eht* or long term responses to external variation such as meals. Now, we turn our attention to using the model to understand and interpret data from the Hashemi Lab on the time course of serotonin in the extracellular space after serotonin release has been stimulated. The Hashemi lab uses Fast Scan Cyclic Voltammetry (FSCV) to measure the time course of *eht* in vivo in different brain regions (see “Methods” section). In this section we examine data recently collected in vivo of responses of male and female mice in the CA2 region of the hippocampus [62]. The medial forebrain bundle was stimulated and the antidromic spikes stimulate the DRN that projects to the hippocampus and releases serotonin into the extracellular space. The serotonin is taken up by the SERTs and Uptake 2. The mean curves of *eht* for 23 male mice and 23 female mice are given by the red dots in Fig. 4a, b. By varying a small number of parameters, we were able to obtain model response curves (blue) that are very similar to the responses in the experimental data. The model curves are given by the corresponding blue curves. The male and female average curves are quite similar although the female curve dips further below baseline. The model parameters that were varied are given in Table 4 and discussed below.

**Table 4:** Parameter values for male and female average curves

Parameter	Meaning	Male	Female
$V_{max}$ of $V_{\mathrm{U2}}$	Strength of Uptake 2	1680	1680
Uptake 2	H = 0 below and 1 above	60.5–75.5	60.5–70.5
slope of *inhib*	strength of $5HT_{1B}$	10	12.5
$\beta _1$	Scale for $G^*_{ht}$ dynamics	0.8	0.85
$\beta _2$	Scale for $T^*_{ht}$ dynamics	0.6	0.7
$\beta _3$	Scale for $B_{ht}$ dynamics	0.8	0.85
*r*	Strength of *fire*(*t*)	18	18.5

It is worthwhile to discuss how the shapes of the curves are influenced by the autoreceptor dynamics. Figure 4c shows the time course of the three variables, $B_{ht}, G_{ht}^*, T_{ht}^*$. The red curve is $B_{ht}$, which is *eht* bound to the $5HT_{1B}$ autoreceptors, and it mimics the *eht* curve as it should. The blue curve, which is the activated G-protein, $G_{ht}^*$, rises more slowly and peaks later than the $B_{ht}$ curve. The black curve, which is the activated regulator of G-protein signaling, $T_{ht}^*$, rises even more slowly and peaks even later. Notice than when *eht* has returned to baseline (at about 12–14 s), $G_{ht}^*$ is still above its baseline and therefore it is still inhibiting release; this is what drives the *eht* curve below baseline. Still later, the increase in $T_{ht}^*$ drives $G_{ht}^*$ down below its baseline so serotonin is being released faster than normal; this causes the *eht* curve to turn upwards towards baseline. Finally, all three variables, $B_{ht}, G_{ht}^*, T_{ht}^*$, relax towards their respective baselines. The parameters $\beta _1$, $\beta _2$, and $\beta _3$ multiply the right hand sides of the differential equations for $G_{ht}^*, T_{ht}^*$, and $B_{ht}$ and speed up or slow down the differential equations depending on whether they are greater than 1 or lower than 1. In the standard model they are all set to 1. These parameters change the shapes of the $G_{ht}^*$ and $T_{ht}^*$ curves and therefore the *eht* curve also. As one can see in Table 4, they are somewhat different for the male and female average curves. The higher female values cause the female average curve to dip further below baseline than the average male curve. Figure 6a shows 17 males response curves, and one can see how different their shapes are, probably because the different animals have different autoreceptor dynamics.

**Fig. 5:**
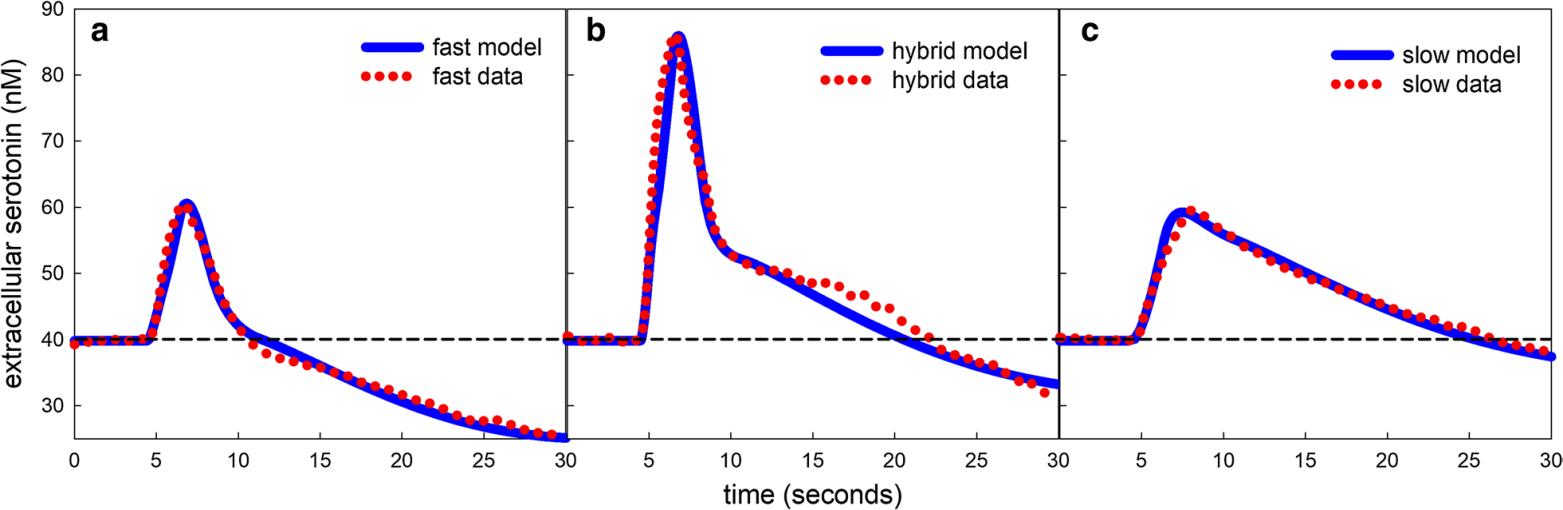
Fast, hybrid, and slow responses in the SNr. **a**, **c** Show simulations of fast and slow responses with experimental data taken from [7]. **b** Shows a simulation and previously unpublished data for a hybrid response. Red dots indicate experimental data and blue curves are model simulations. All responses were in the SNr. The dashed horizontal line is the average baseline concentration of *eht* in the SNr. Parameters for the simulations are shown in Table 5 and the significance of the parameters is discussed in the text

**Fig. 6:**
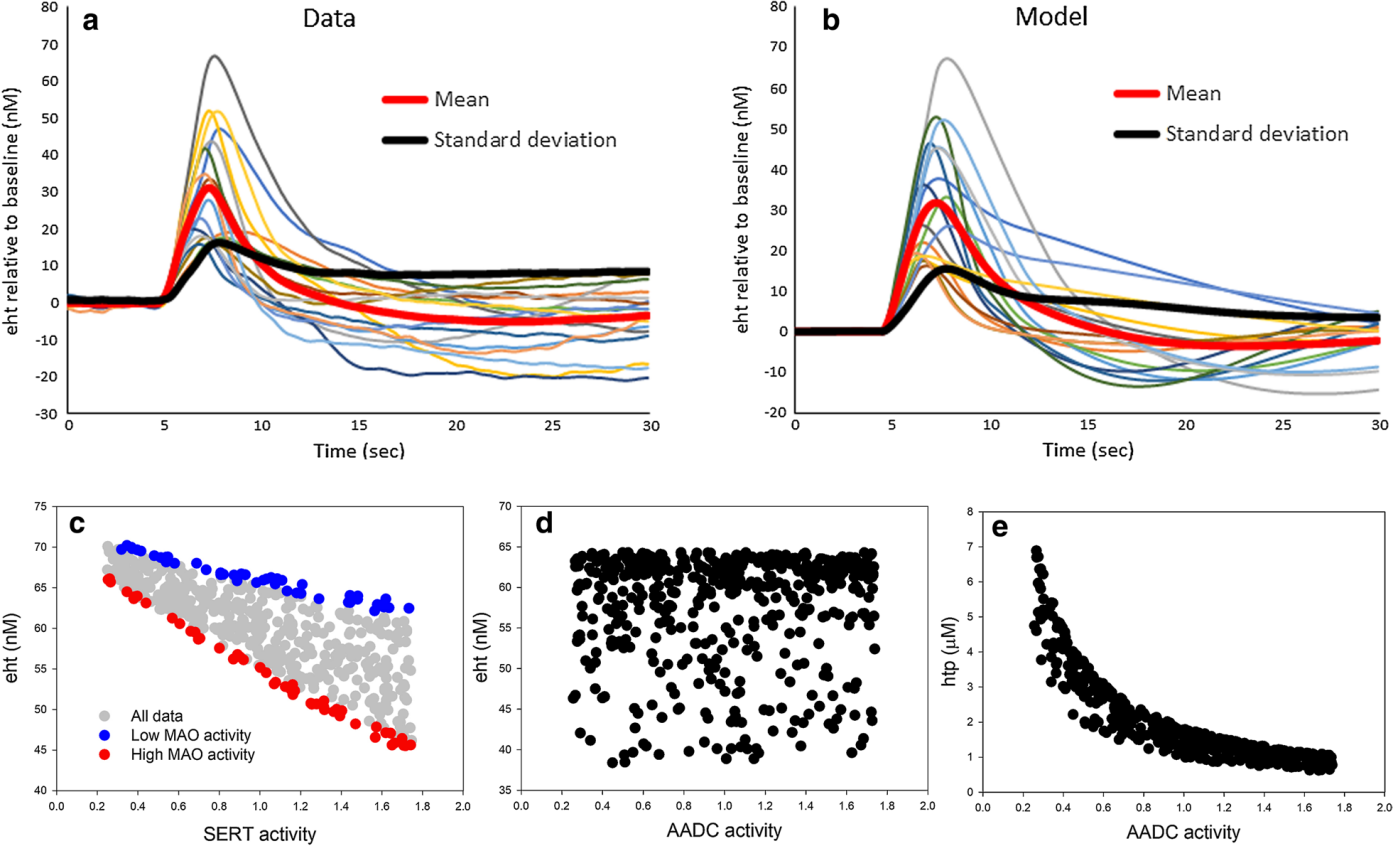
Investigations using the systems population model. **a** Shows the time courses of *eht* in the hippocampus of 17 male mice after 2 s of stimulation at $t = 5$ s (Hashemi Lab). The thick red and black curves are the time courses of the mean and standard deviation, respectively. The response curve are diverse and have different heights, peaks and shapes. **b** Shows 17 randomly selected response curves in a systems population model of 1000 individuals with 40% independent variation in many parameters in the model (see text in “Variability in response to stimulation” section for details). The red and black curves are the time courses of the mean and the standard deviation of the 1000 model individuals, respectively. **c** Shows a systems population model of 500 individuals where the expression values of SERT and MAO were varied from 25 to 175% of normal and all other constants were fixed. The blue dots are individuals with low MAO activity and the red dots are individuals with high MAO activity. SSRIs have a greater effect on individuals with high MAO activity. **d**, **e** The results from a systems population model (500 individuals) where blood tryptophan and the expression level of AADC were varied from 25 to 175% of normal and all other constants were fixed. **d** Shows that *eht* is uncorrelated to AADC activity. **e** Shows that as AADC activity goes down *htp* concentration goes up so the flux through AADC remains nearly constant. This may explain why supplementation by vitamin B6 (a co-factor for AADC) is an ineffective treatment for depression

We determined the value of the $V_{max}$ of Uptake 2 in the normal model by requiring that the distribution of values of *eht* in the systems population model be similar to experimental distributions (see Fig. 2) measured in the hippocampus. But we expect that in some nuclei and some experiments the $V_{max}$ of Uptake 2 will be much larger depending on the density of glial cells, the prevalence of OCT, DA, and NET transporters and the proximity of the measuring electrode to the glial cells. We needed the high values here to bring down the model curve from the peak rapidly as is seen in the experimental data. Uptake 2 is zero below the lower number of the H range and equals one above the upper number and increases linearly in between. The slope of $inhib_{ht}$ represents how sensitive the inhibition of release is to the concentration of $G_{ht}^*$. So the female autoreceptors not only respond more quickly but inhibit release more strongly (see Table 4). The parameter *r* is proportional to how much serotonin is released after stimulation of the MFB. All other parameters were as in the standard normal model described in the Methods. It is not surprising that the average response curves are different for males and females and that the best fit parameters are different because estradiol affects both TPH expression and a variety of 5HT receptors [84–86].

### Two serotonin uptake mechanisms and the effect of histamine

In 2014 we published experimental and mathematical results that altered our view of serotonin clearance after stimulation in two major ways [7]. In the experiments, the medial forebrain bundle (MFB) was stimulated. The antidromic spikes stimulated the DRN and the serotonin concentration was measured in vivo in the substantia nigra pars reticulata (SNr) of mice. The first major observation was that the serotonin concentration first went up, but then descended below baseline in almost all experiments (see Figs. 4a, b,  5a, b). As discussed in “The dynamics of the autoreceptors” section, this showed that the effect of the $5HT_{1B}$ autoreceptors is long-lasting (30 s to 1 min) because they still inhibit release after the *eht* concentration has returned to normal and this drives the *eht* concentration below baseline. In our 2010 model [1], the autoreceptor effect was instantaneous in that *eht* inhibited release when it was above baseline and stimulated release when it was below. The new experimental evidence showed that this was wrong and led directly to the new $5HT_{1B}$ autoreceptor model in this paper.

The second major finding in [7] was that there were clearly two different uptake mechanisms at work. Consider the experimental curves (red dots) in the three panels of Fig. 5; each curve is the average of 5 animals. The slope of the *eht* curve is the rate of uptake and one can see that there are two distinct slopes. In some measurements (Fig. 5a), the *eht* curve descended rapidly to baseline; we call these responses “fast.” In some measurements (Fig. 5c), the *eht* curve descended slowly and linearly to baseline; we call these responses “slow.” And, in some experiments (Fig. 5b), the *eht* curve descended rapidly for a while but then switched to descending slowly; we call these responses “hybrid.” The majority of experimental responses were hybrid. As explained in the Methods, we hypothesized that the “fast” uptake transports serotonin into glial cells via the dopamine transporter (DAT), the norepinepherine transporter (NET), and the organic cation transporter (OCT). This is what we call Uptake 2, and the fact that most response curves were “hybrid” suggested that Uptake 2 normally operates only above a concentration that is higher than the steady state level of *eht*. We assume that the “slow” uptake is via SERTs.

In [7], we used a very simple mathematical model to help explain the data. It had a single differential equation for *eht* with two uptakes and a release term multiplied by a function of time $1 -A(t)$ where *A*(*t*) represented the “strength” of the autoreceptor effect as a function of time. We found that if we chose *A*(*t*) appropriately, we could match different fast. slow, and hybrid responses. Thus, there was no internal chemistry of the serotonin neuron and the autoreceptor effect was put in by hand. Our purpose here is to show that our new full serotonin model with its complicated autoreceptor dynamics could also match the varied responses, fast, slow, hybrid in the SNr. However, when we ran the model the *eht* curves would go below baseline and then curve up towards baseline (like the male and female average curves in Fig. 4 or the male experimental curves in Fig. 6a), because once $T^*$ drives $G^*$ below baseline extra serotonin is released. But the experimental curves in the SNr (Fig. 5) do not curve up, which was a clear indication that something else was going on.

In [87], it was shown that when the MFB is stimulated, then not only is serotonin released in the SNr, but histamine is also released. Furthermore, there was early experimental evidence that histamine can inhibit serotonin release [88, 89] and this was confirmed by our study [8] in 2016. Finally, in 2017 we published a full mathematical model of a histamine neuron and that model included the dynamics of the $H_3$ autoreceptor for histamine [54]. It is known [89] that $H_3$ autoereceptors do occur on serotonin varicosities in the SNr, so we included an $H_3$ receptor for histamine on our serotonin varicosity (see Fig. 1 and “Methods” section). We do not have the time course of histamine in the SNr because in 2014 the measurement techniques were not yet developed. So we will take our histamine time course in the extracellular space, *eha*, from the control and model curves in Fig. 5 of [54]. Note how complicated the dynamics of *eht* are. When one stimulates the MFB, serotonin is released into the extracellular space stimulating dynamical changes in the $5HT_{1B}$ autoreceptor variables, $B_{ht}$, $G^*_{ht}$, $T^*_{ht}$. However, histamine also increases in the extracellular space stimulating dynamical changes in the $H_3$ receptor variables, $B_{ha}$, $G^*_{ha}$, $T^*_{ha}$. Both of the activated G-proteins, $G^*_{ht}$ and $G^*_{ha}$ inhibit serotonin release via the functions $inhib(G^*_{ht})$ and $inhib_{ha}(G^*_{ht})$, respectively; see the differential equation for *eht*.

Surprisingly, it was quite easy to give adjustments for a small number of parameters (Table 5) that distinguish between fast, slow, and hybrid responses. First, we raised the $V_{max}$ of SERT from 250 to 433 and this lowered the steady-state value of *eht* from 60 nM to 39.8 nM consistent with the measurements in [60]. Note that the slope of the slow parts of the experimental curves (red dots) in Fig. 5a–c are very similar, so it is not surprising that the $V_{max}$ of 433 for SERT worked in all three cases (Table 5).

**Table 5:** Parameter values for fast, hybrid, and slow

Parameter	Meaning	Fast	Hybrid	Slow
$V_{max}$ of $V_{\mathrm{SERT}}$	Strength SERT	433	433	433
$V_{max}$ of $V_{\mathrm{U2}}$	Strength of Uptake 2	3220	5600	1400
Uptake 2	H = 0 below and 1 above	40–50	52–62	55–65
Slope of *inhib*	Strength of $5HT_{1B}$	(.2)(12.5)	(.1)(12.5)	(.1)(12.5)
Slope of *inhibsyn*	Strength of $5HT_{1B}$	(.2)(12.5)	(.1)(2.5)	(.1)(2.5)
Slope of $inhib_{ht}$	Strength of $H_3$	5	3	2
*r*	Strength of *fire*(*t*)	10.3	22	4.5

We discuss each of the other relevant parameters in turn. The $V_{max}$ of Uptake 2 indicates its strength. It is quite consistent for repeated measurements of one animal but varies widely between animals almost certainly because it depends on the geometry of electrode placement relative to serotonin varicosities and glial cells. Uptake 2 is zero below the lower number of the H range and equals one above the upper number and increases linearly in between. The size of this $V_{max}$ is determined by the rapidly decreasing portion of the curve after the peak for Fig. 5a, b. Uptake 2 has little effect on the slow response in Panel c because the curve peaks at 59 nM and Uptake 2 does start affecting uptake until the concentration is above 55nM. The appropriate Uptake 2 transition range is determined roughly by the transition from fast decrease to slow decrease.

The slopes of three *inhib* functions represent how sensitive the inhibition of release or synthesis is to the concentrations of *eht* and *eha*, respectively. To fit this data we had to greatly weaken the sensitivity of the two serotonin functions, *inhib* and *inhibsyn*, and the strength of the inhibition of serotonin release by the $H_3$ receptors varied in the three cases as indicated in Table 5. The parameter *r* is proportional to how much serotonin is released by the stimulation. In our modeling we’ve found that *r* is consistent with repeated measurements on one animal but varies widely between animals. This is not surprising since electrode placement differs between animals as does the stimulation of the MFB. All other parameters in the model were as in the normal standard model described in the Methods.

The modeling shows that the main differences between fast, slow, and hybrid depend on the transition region for the function *H* that governs the concentration range where Uptake 2 becomes functional. It is particularly gratifying that Threlfell et al. [89] found that not only does histamine play a major role in regulating serotonin in the SNr, but they also found that there are very few $5HT_{1B}$ autoreceptors on serotonin varicosities in the SNr, which corresponds exactly to what we found by modeling (the (.1) and (.2) multipliers for the slopes of $inhib_{ht}$ and $inhib_{htsyn}$).

We note that the response curves in Fig. 5 look quite different from the response curves in Fig. 4 where the 5HT concentration drops below baseline and then bends back toward baseline within 25 s after stimulation. There are two reasons for the difference. First, the measurements are in two different brain nuclei, the CA2 region of the hippocampus in Fig. 4 and the SNr in Fig. 5. We expect that the response curves after stimulation will be quite different in different regions because SERT and autoreceptor densities vary between regions and the speed of the autoreceptor effect will vary depending on G-protein concentrations. The second, and probably more important reason, is that when the MFB is stimulated HA as well as 5HT is released into the SNr and HA binds to $H_3$ receptors on 5HT terminals and inhibits 5HT release. The time course of that inhibition depends on the dynamics of the G-proteins associated to the $H_3$ receptor, and it is that HA inhibition that causes the response curves in Fig. 5 to keep descending during the first 25 s after stimulation.

### Variability in response to stimulation

In “The dynamics of the autoreceptors” section, we used the deterministic model to fit the mean *eht* response curves for male and female mice in the hippocampus. We showed that small changes of parameters allowed us to fit both mean curves in response to 2 s of stimulation at $t=5 s$. In this section, we confront the variability in the male curves themselves. Figure 6a shows the responses of the 17 male mice. The experimental responses are measured and graphed for each mouse relative to the baseline level of *eht* that is represented in Panel a by $eht =0.$ One can see how large the variation is. The curves peak at different times and at different heights. Most, but not all, of the curves descend below baseline and their shapes are quite different; some continue descending while others reach a minimum and then rebound towards zero. The thick red curve is the mean and the thick black curve is the standard deviation, which is substantial even between 15 and 30 s.

We investigated what variation in the main parameters of the model would be necessary to obtain the variation seen in the experiments. To do this we created a virtual population of 1000 individuals with the independent variations in parameters. The following parameters were varied uniformly from 40 below to 40% above their normal values: the $V_{max}$ values for $V_{\mathrm{AADC}}$, $V_{\mathrm{CATAB}}$, $V_{\mathrm{MAT}}$, $V_{\mathrm{SERT}}$, $V_{\mathrm{TPH}}$, $V_{\mathrm{U2}}$; the slope of *inhib* and *inhibsyn*; *eha*, the concentration of histamine in the extracellular space, and $\beta$ that controls the speed of the autoreceptors. In addition, we varied the the parameter *r* in *fire*(*t*) by 25% and the time of the peak by 20%.

Figure 6b shows a random sample of 17 of the 1000 model male curves. The thick red curve is the mean of the 1000 model curves and the thick black curve is the standard deviation. The mean curve matches the experimental mean curve very well. The model standard deviation curve is very close to the experimental standard deviation except that at long times (20 to 30 s) it descends slightly while the experimental standard deviation remains constant. Overall, one can see visually that the 17 model curves and the 17 experimental curves look similar as groups of curves. For each of the 1000 individuals, we record their steady state values as well as the values of all of their parameters so we can use multi-linear regression to find which parameters contributed most to the variation in the response curves. At $t = 7s$ (roughly the time of the peak), the three variables that contributed most, in order, were the strength of fire(t), the timing of the peak in fire(t), and the $V_{max}$ of the SERTs. At $t=15 s$ (when most of the curves have returned to near baseline), the three parameters that contributed most to the variation in response were the $V_{max}$ of TPH, the speed of the autoreceptors, and the $V_{max}$ of MAT.

The purpose of autoreceptors is to stabilize the concentration of *eht* in the extracellular space. Thus, one would expect that if the autoreceptors were stronger (larger *s*) then one would need larger variation in parameters to obtain the variation in the experimental curves, and conversely, if the autoreceptors were weaker, then less variation in parameters would be needed to obtain the experimental variation. Indeed, this is true (simulations not shown).

### Using population models to understand drug efficacy and clinical measurements

It is known that the expression levels of most enzymes can vary by about 25% or more between individuals [14–16]. This means that the $V_{max}$ values of all the enzymes and transporters in our model vary by at least 25% and that any population of individuals will express this diversity. This poses large issues for drug discovery and treatment because it means that different individuals will react very differently to drugs, as is well-known [90–92]. In “Variability in response to stimulation” section, we used our systems population model to investigate how much variation in many variables of the model is necessary to obtain the observed variation in experimental response curves. In this section, we give two brief, simple examples that show how to use variation in a small number of variables to investigate questions about drug efficacy and clinical measurements.

It is well known that most antidepressants have limited therapeutic benefits for many patients [93, 94], and it is therefore important to find what patient characteristics lead to greater efficacy. In Fig. 6c, we show results from our systems population model where we varied only two constants, the expression level ($V_{max}$) of SERT and the expression level of MAO, from 25 to 175% of normal. Each dot is an individual in a population of 500. The y-axis is the concentration of *eht*, extracellular serotonin, and the x-axis is the expression level of SERT. The blue dots are the individuals with low MAO activity and the red dots are individuals with high MAO activity. The conclusion is clear. Blocking SERTs with an SSRI (equivalent to lowering the expression level) will have a much greater effect on individuals with high MAO activity than on individuals with low MAO activity. Therefore, the systems population model suggests that it is high MAO individuals that will benefit the most from an SSRI.

Next, aromatic amino acid decarboxylase (AADC) is a key enzyme on the synthesis pathway of serotonin and AADC requires vitamin B6 as a cofactor. This raises the question of whether vitamin B6 status could be correlated with depression. In fact, correlations have been found [95–97] and vitamin B6 supplementation has been suggested for depression. However, the clinical results have been disappointing [98]. Why? To investigate this question, we varied blood tryptophan and the $V_{max}$ of AADC from 25 to 175% in our systems population model. We already showed in “The effect of tryptophan input” section that *eht* depends in increasing fashion on blood tryptophan, so we concentrate on AADC here. Figure 6d shows that *eht* is completely uncorrelated with AADC activity and panel e shows why. As AADC activity goes down, its substrate, *htp*, builds up and this compensates for the reduction in expression level of AADC because the normal concentration level of *htp* is way below the $K_m$ of AADC (see Table 2). Thus, deficiency or excess of the vitamin B6 co-factor does not change the flux, $V_{\mathrm{AADC}}$, very much. This explains why supplementary vitamin B6 has not been a successful treatment for depression.

## Conclusions

We have created a new model of serotonin metabolism including transport of tryptophan from the blood, synthesis of serotonin, packaging into vesicles, release, reuptake and control by autoreceptors. We have shown that the models can be used to investigate the distribution of values of serotonin in the extracellular space and the effects of tryptophan input and meals. We have demonstrated how the models can be used to understand the behavior of response curves in the extracellular space in different brain nuclei. We used the systems population model to investigate the origins of the variation in response curves in different animals, and we showed how the systems population model can be used in drug discovery and to understand clinical measurements. The codes for both the deterministic model and the systems population model are available from the authors and can be used by other researchers to investigate the serotonergic system.

## Data Availability

Experimental data is available from the Hashemi Lab at the University of South Carolina. Codes for the deterministic model and the systems population model are available from HFN and MR at Duke University.
